# Fractured metallic tracheostomy tube in a child: a case report and review of the literature

**DOI:** 10.1186/1752-1947-4-234

**Published:** 2010-08-02

**Authors:** Patorn Piromchai, Piyawadee Lertchanaruengrit, Patravoot Vatanasapt, Teeraporn Ratanaanekchai, Sanguansak Thanaviratananich

**Affiliations:** 1 Department of Otorhinolaryngology, Faculty of Medicine, Khon Kaen University, 40002, Thailand; 2 Department of Pediatric, Vibhavadi Hospital, Bangkok, 10900, Thailand

## Abstract

**Introduction:**

Tracheostomy is a common airway procedure for life support. The fracture of the tracheostomy tube is a rare complication. We report a case of a 14-year-old boy whose fractured stainless steel tracheostomy tube dislodged into the tracheobronchial tree. We include a literature review and proposed recommendations for tracheostomy care.

**Case presentation:**

A 14-year-old Thai boy who had a stainless steel tracheostomy tube presented with a complaint of intermittent cough for 2 months. During tracheostomy tube cleaning, his parents found that the inner tube was missing. A chest X-ray revealed a metallic density foreign body in his right main bronchus. He underwent bronchoscopic removal of the inner tracheostomy tube and was discharged without further complications.

**Conclusion:**

A fractured tracheostomy tube is a rare complication. Appropriate cleaning and scheduled replacement of the tracheostomy tube may prevent this complication.

## Introduction

Tracheostomy is a common airway procedure for life support. Across the United States of America the tracheostomy rate ranges from 150 to 300 per 100,000 patients discharged from hospital; the pediatric tracheostomy rate is 7.5 per 100,000 [1]. The procedure is safe and the mortality rate is less than 5% [2] and the complications can be categorized as early or late complications. The early complications are hemorrhage, pneumothorax, obstruction of the tracheostomy tube and wound infection. The late complications are granulation formation, airway scarring, erosion of the innominate artery and tracheoesophageal fistula. Fracture of a metallic tracheostomy tube is a rare complication.

We report a case of a 14-year-old boy with a fractured metallic tracheostomy tube in the tracheobronchial tree. We also include a review of the literature and the proposed the recommendations for tracheostomy care.

## Case presentation

A 14-year-old Thai boy presented to the community hospital with a complaint of intermittent cough of two weeks duration. Four years previously, he had undergone a tracheostomy for laryngeal stenosis following prolonged intubation after a burr-hole craniotomy for subdural hematoma evacuation. A No. 5 stainless steel tracheostomy tube was put in place. The current tracheostomy tube had been used for one year.

Two months previously, the patient started coughing and during the daily cleaning session his parents found that the inner tube was missing. He was brought to the family physician immediately. The patient was diagnosed with acute bronchitis and a new tracheostomy tube of the same size was inserted. After discharge, the parents reported that their child still coughed off and on every week. He slept well during the night without any breathing difficulties and had no abnormal breath sounds.

One day prior to admission, the boy had more severe and persistent cough. He was sent to the community hospital again. The chest X-ray revealed a metallic density foreign body in his right main bronchus. Subsequently, he was referred to our university hospital for definite treatment.

**Figure 1:**
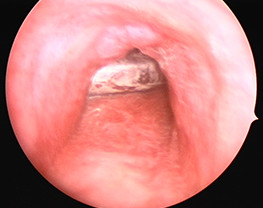
**Bronchoscopic view of the foreign body in the right main bronchus**.

**Figure 2:**
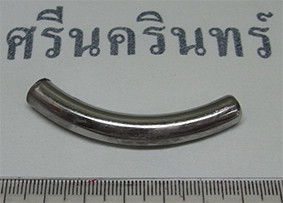
**Part of inner tracheostomy tube that dislodged into the right main bronchus**.

**Figure 3:**
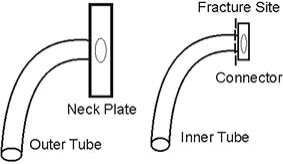
**The fracture site at the junction between the inner tube and connector**.

On arrival, the patient had occasional cough with hyperpnea. His vital signs were: a body temperature of 38.0° Celsius; a pulse rate of 140 beats per minute; respiratory rate of 44 times per minute; and blood pressure of 120/80 mmHg. The chest auscultation revealed decreased breath sounds on the right side but no chest wall retraction. An X-ray of the chest was performed. Patchy infiltration of the right lower lung and a metallic foreign body in the right main bronchus were found. He was transferred to the operating room for bronchoscopic removal under general anesthesia. The foreign body (inner tube of the previous tracheostomy tube) was retrieved from the right main bronchus and removed through the tracheostomy stoma (Figure 1). A fracture at the junction between the inner tube and connector was found (Figures 2 and 3). His pneumonia was treated with intravenous amoxicillin with clavulanic acid for three days before switching to oral form for 11 days. A follow-up chest X-ray showed decreased infiltration compared with the prior film. He was discharged with improvement of his symptoms. He had fully recovered at the one month follow-up and there were no signs of any late complications.

## Discussion

A fractured tracheostomy tube is a rare complication. Patients are usually misdiagnosed as having asthma, chronic bronchitis or pneumonia before the definite diagnosis is made. The first case report of a fractured tracheostomy tube was in 1960 by Bassoe and Boe [3]. Since then, this complication has been published in medical literature from time to time. We reviewed 20 cases from 18 published reports. There were 15 males (75%) and four females (20%). Fourteen metallic tubes and three polyvinyl chloride (PVC) tubes were reported. The most common dislodged sites were the trachea and the right main bronchus. The most common fracture was at the junction between the tube and the neck plate (Table 1).

**Table 1:** Summary of previous case reports.

Authors	Year	Sex	Age	Material	Lodging site	Fracture site
Bassoe and Boe [3]	1960	F	35	Metal (silver and nickel)	RMB	Distal end of cannula

Kakar and Saharia [15]	1972	M	40	Metal (copper and zinc)	T and LMB	Junction between tube and neck plate

Kemper *et al*. [4]	1972	M	48	Metal	T and RMB	Inner tracheostomy tube

Sood [7]	1973	M	60	PVC	T	Junction between tube and flange

Maru *et al*. [5]	1978	M	50	Metal	T and LMB	Junction between tube and neck plate

Gupta and Chhangani [18]	1981	M	15	Metal	LMB	Flange

Gupta and Chhangani [18]	1981	M	ND	Metal	RMB	Flange

Bhalla [19]	1983	F	50	ND	LMB	Outer tube

Okafor [8]	1983	M	40	Metal (silver and Zinc)	T and RMB	Junction between tube and neck plate

Bowdler and Emery [9]	1985	M	3	Silver	T and RMB	Junction between tube and neck plate

Bowdler and Emery [9]	1985	M	76	Silver	C and RMB	Junction between tube and neck plate

Otto and Davis [20]	1985	ND	3	Stainless steel	T and RMB	Junction between tube and neck plate

Majid [10]	1989	F	63	Silver	T and LMB	Junction between tube and neck plate

Ming [21]	1989	M	50	Silver	RMB	Junction between tube and flange

Gupta and Ahluwalia [11]	1996	M	10	Metal	RMB and LPBS	Flange

Krempl and Otto [14]	1999	M	48	ND	T and RMB	Fenestra

Gana and Takwoingi [12]	2000	M	7	PVC	RMB and LMB	ND

Srirompotong and Kraitakul [13]	2001	M	7	ND	LMB	Inner tracheostomy tube

Wu [22]	2007	F	14 months	PVC	T and LMB	ND

Radpay [23]	2009	M	41	Metal	T and LMB	Shaft

RMB = right main bronchus; LMB = left main bronchus; LPBS = left posterior basal segment; T = trachea; C = carina; ND = no data; PVC = polyvynylchloride.

Tracheostomy tubes are made from metal, PVC or silicone. Most plastic pediatric tubes are disposable and cannot be reused. The metallic tubes are more suitable for prolonged use as they are unlikely to fracture and can be washed and boiled. Traditional metallic tracheostomy tubes are made from silver, steel, copper or zinc, all of which are prone to corrosion by alkaline tracheal secretion [4]. In the modern era, metallic tracheostomy tubes are made from stainless steel which contains steel and chromium. Stainless steel does not stain, corrode or rust as easily as ordinary steel.

The weak points of the tracheostomy tube are the junctions between the tube and the neck plate, the distal end of the tube and the fenestration site [5-10]. We reported a case of a fracture at the junction between the inner tube and connector, which is a rare fracture site. Prolonged wear, ageing of the tubes and repeated sterilization have been proposed as risk factors of a fractured tracheostomy tube [8,11-14]. Alkaline bronchial secretion, tissue reactivity from plastic tubes, long continued high internal stresses on the surface and manufacturing defects were also reported as causes of this complication [11-13]. In our opinion, the fracture of the tracheostomy tube in this patient may have been due to prolonged wear and ageing of the tube. Loss to follow-up is a common problem in many reports [8-10,13-15]. The cause of late complications may be due to a lack of periodical check-ups for signs of wear and tear or review of the tracheostomy care, including fracture of the tracheostomy tube.

Fractured tracheostomy tubes dislodged into the tracheobronchial tree may produce acute and chronic respiratory symptoms. Presenting symptoms, such as choking and dyspnea, were observed in this group, but children with delayed diagnosis have milder symptoms such as coughing and wheezing [16]. Delayed diagnosis can result in problems such as prolonged cough and wheezing, pneumonia and bronchiectasis. In one study, the duration of the symptoms ranged from one to 132 months (median three months) [17]. Our patient had experienced symptoms for two months. One should suspect foreign body aspiration in children with persistent respiratory symptoms, especially those who have a risk factor for aspiration.

Tracheostomy care is a crucial step in the prevention of this complication. There is no current consensus on tracheostomy tube care. From the previous report and our experience, we suggest the following recommendations:

1. Change the tracheostomy tube every six months [13,14].

2. Clean the inner cannula daily or every other day [13,14]. More frequent cleaning may be required depending upon the amount and nature of the patient's secretions.

3. Daily dressing of the tracheostomy site [14].

4. Tube ties should be changed weekly [14].

5. Patients should be provided with two sets of inner tracheostomy tubes at home. Alternative use of these sets may reduce wear and tear of the tube [8,14].

6. Regular check-ups are important. Follow-up systems should be established in any hospital that is involved in caring for patients who undergo a tracheostomy.

7. Patients and caregivers should be properly trained in the care of tracheostomy patients and the complications that could occur. A periodic review of the techniques may be helpful.

8. In the case of an emergency, immediate hospital contact and a good referral system are critical for the early detection and management of these complications.

## Conclusion

Fracture of the metallic tracheostomy tube is a rare complication and may be overlooked. This case involved a fracture at the junction of the inner tube and connector. Appropriate cleaning and scheduled replacement of the tracheostomy tube may have prevented this complication.

## Consent

Written informed consent was obtained from the patient's mother for the publication of this case report and accompanying images. A copy of the written consent is available for review by the Editor-in-Chief of this journal.

## Competing interests

The authors declare that they have no competing interests.

## Authors' contributions

PP analyzed and interpreted the patient's data and was a major contributor to the manuscript. PL analyzed the patient's data and wrote the discussion section. PV performed the operation, collected and interpreted the patient's data. TR is the attending physician and collected the data. ST analyzed the patient's data and revised the manuscript. All authors read and approved the final manuscript.
